# Supplementary material for: FDXR-Associated Oculopathy: Congenital Amaurosis and Early-Onset Severe Retinal Dystrophy as Common Presenting Features in a Chinese Population
Source: Genes (Basel). 2023 Apr 21;14(4):952. doi: 10.3390/genes14040952 (PMC10137360; doi:10.3390/genes14040952)
Supplement: Supplementary file 1 [file genes-14-00952-s001.zip › YiST-FDXR-Supplementary Table S2.pdf]

Supplementary Table S2. The classification of variants in FDXR identified in our study based on ACMG/AMP criteria.

Variant ID	Nucleotide change	Effect	ACMG/AMP Evidence	Classification
M1	c.578G>A	p.R193H	PS1, PS4, PM5, PP1, PP2, PP3, PP4	P
M2	c.235C>T	p.R79C	PS4, PP1, PP2, PP3, PP4	LP
M3	c.310C>T	p.R104C	PS4, PP1, PP2, PP3, PP4	LP
M4	c.400G>A	p.G134R	PS4, PP1, PP2, PP3, PP4	LP
M5	c.917G>A	p.R306H	PS4, PM5, PP1, PP2, PP4	LP
M6	c.463C>T	p.R115G	PS4, PP2, PP3, PP4	LP
M7	c.698A>G	p.Q233R	PS4, PP2, PP3, PP4	LP
M8	c.682C>T	p.R228W	PS4, PM5, PP2, PP3	LP
M9	c.940G>T	p.V314L	PS1, PS4, PM5, PP1, PP2, PP4	P
M10	c.343A>G	p.R115G	PM2, PP1, PP2, PP4	LP
M11	c.974G>A	p.G325D	PM2, PP1, PP2, PP3, PP4	P
M12	c.695T>C	p.L232P	PM2, PP1, PP2, PP3, PP4	P
M13	c.823C>T	p.R275W	PS1, PS4, PM5, PP1, PP2, PP3, PP4	P
M14	c.1194G>T	p.W398C	PM2, PP1, PP2, PP3, PP4	P
