# Supplementary material for: FDXR-Associated Oculopathy: Congenital Amaurosis and Early-Onset Severe Retinal Dystrophy as Common Presenting Features in a Chinese Population
Source: Genes (Basel). 2023 Apr 21;14(4):952. doi: 10.3390/genes14040952 (PMC10137360; doi:10.3390/genes14040952)
Supplement: Supplementary file 1 [file genes-14-00952-s001.zip › YiST-FDXR-Supplementary Table S3.pdf]

Supplementary Table S3. Biallelic potential pathogenic *FDXR* variants in this and previous studies

Nucleotide	Effect	Bioinformatic prediction tools						gnomAD	Reported
change		CADD	REVEL	SIFT	Polyphen2	PROVEAN	fathmm MKL coding	Frequency	in
c.1 A>G	p.M1?	21.9	/	/	/	/	/	/	Previous study
c.1058G>A	p.Cys353Tyr	27.2	0.620	DA	PRD	DE	DE	6/282632	Previous study
c.1102G>A	p.Asp368Asn	24.8	0.153	DA	POD	DE	DE	2/251302	Previous study
c.1115C>A	p.Pro372His	26.2	0.492	DA	POD	DE	DE	4/282602	Previous study
c.1156C>T	p.Arg386Trp	32.0	0.530	DA	PRD	DE	DE	45/250556	Previous study
c.1189G>A	p.Gly397Ser	28.8	0.842	DA	PRD	DE	DE	10/278128	Previous study
c.1194G>T	p.Trp398Cys	31	0.533	DA	PRD	DE	DE	/	/
c.1208C>T	p.Pro403Leu	27.6	0.462	DA	PRD	DE	DE	3/248366	Previous study
c.1255C>T	p.Glu419*	41.0	/	/	/	/	/	/	Previous study
c.1279G>C	p.Ala427Pro	20.2	0.132	T	B	N	N	2/249648	Previous study
c.1309G>A	p.Gly437Ser	25.5	0.206	DA	POD	DE	DE	/	Previous study
c.1309G>T	p.Gly437Cys	25.9	0.380	DA	PRD	DE	DE	/	Previous study
c.1343G>A	p.Arg448Gln	32.0	0.243	DA	PRD	DE	DE	2/246380	Previous study
c.1429 G>A	p.Glu477Lys	24.7	0.220	DA	PRD	DE	DE	/	Previous study
c.151T>C	p.Phe51Leu	25.0	0.585	DA	PRD	DE	DE	/	Previous study
c.221C>T	p.Pro74Leu	25.5	0.596	DA	PRD	DE	DE	4/250916	Previous study
c.235C>T	p.Arg79Cys	29.0	0.653	DA	PRD	DE	DE	2/250924	This study
c.250C>T	p.Pro84Ser	24.1	0.766	DA	PRD	DE	DE	/	Previous study
c.310C>T	p.Arg104Cys	31.0	0.517	DA	PRD	DE	DE	16/281296	This study
c.325G>A	p.Gly109Ser	28.5	0.447	DA	PRD	DE	DE	1/250090	Previous study
c.332T>C	p.Val111Ala	26.8	0.568	DA	PRD	DE	DE	/	Previous study
c.343A>G	p.Arg115Gly	23.6	0.409	T	B	DE	DE	/	This study
c.35C>G	p.Ser12Trp	23.2	0.104	DA	POD	N	N	/	Previous study
c.400G>A	p.Gly134Arg	29.7	0.818	DA	PRD	DE	DE	1/249762	This study
c.427A>T	p.Ile143Phe	22.7	0.199	DA	PRD	DE	DE	/	Previous study
c.461C>T	p.Ala154Val	24.4	0.562	DA	PRD	DE	DE	/	Previous study
c.463C>T	p.Arg155Trp	24.4	0.545	DA	PRD	DE	DE	4/282390	This and previous study
c.472G>A	p.Val158Met	26.2	0.456	DA	PRD	DE	DE	2/250992	Previous study
c.564_575del12	p.Leu189_Ala192del	/	/	/	/	/	/	/	Previous study
c.577C>T	p.Arg193Cys	27.0	0.841	DA	PRD	DE	DE	5/248686	Previous study
c.578G>A	p.Arg193His	31.0	0.896	DA	PRD	DE	DE	4/248218	This and previous study
c.613A>G	p.Thr205Ala	26.5	0.757	DA	PRD	DE	DE	/	Previous study
c.614C>T	p.Thr205Met	29.8	0.736	DA	PRD	DE	DE	3/251230	Previous study
c.616G>C	p.Asp206His	32.0	0.818	DA	PRD	DE	DE	/	Previous study
c.619A>T	p.Ile207Phe	29.5	0.820	DA	PRD	DE	DE	/	Previous study
c.632C>T	p.Ala211Val	29.1	0.387	DA	POD	DE	DE	/	Previous study
c.643C>G	p.Leu215Val	31.0	0.534	DA	PRD	DE	DE	/	Previous study
c.682C>T	p.Arg228Trp	32	0.793	DA	PRD	DE	DE	4/251200	Previous study
c.683G>T	p.Arg228Leu	32.0	0.821	DA	PRD	DE	DE	4/251208	Previous study
c.695T>C	p.Leu232Pro	29.8	0.776	DA	POD	DE	DE	/	This study

c.698A>G	p.Gln233Arg	26.5	0.604	DA	POD	DE	DE	2/251196	This study
c.724C>T	p.Arg242Trp	32.0	0.559	DA	PRD	DE	DE	1/31338	Previous study
c.820A>T	p.Lys274*	44.0	/	/	/	/	/	/	Previous study
c.823C>T	p.Arg275Trp	24.3	0.336	DA	PRD	DE	DE	2/221422	This and previous study
c.916C>T	p.Arg306Cys	24.7	0.208	DA	POD	DE	DE	7/241968	Previous study
c.917G>A	p.Arg306His	22.8	0.196	T	B	DE	DE	2/242172	This study
c.925C>T	p.(Arg309*)1	41.0	/	/	/	/	/	/	Previous study
c.929delG	p.S316Tfs*19	/	/	/	/	/	/	/	Previous study
c.940G>T	p.Val314Leu	22.5	0.130	T	B	N	DE	18/274502	This and previous study
c.974G>A	p.Gly325Asp	24.1	0.213	DA	B	DE	DE	2/251196	This study
c.979C>A	p.Arg327Ser	25.3	0.350	DA	POD	DE	DE	/	Previous study

Note: REVEL = Rare Exome Variant Ensemble Learner, CADD = Combined Annotation Dependent Depletion, SIFT = Sorting Intolerant From Tolerant, PolyPhen-2 = Polymorphism Phenotyping version 2, PROVEAN = Protein Variation Effect Analyzer, fathmm-MKL = Functional Analysis through Hidden Markov Models - Multiple Kernel Learning, DA=Damaging, T=Tolerated, PRD=Probably damaging, B=Benign, POD=Possibly damaging, DE=Deleterious, N=Neutral, DC=Disease causing
