# Supplementary figures and images for: FDXR-Associated Oculopathy: Congenital Amaurosis and Early-Onset Severe Retinal Dystrophy as Common Presenting Features in a Chinese Population
Source: Genes (Basel). 2023 Apr 21;14(4):952. doi: 10.3390/genes14040952 (PMC10137360; doi:10.3390/genes14040952)

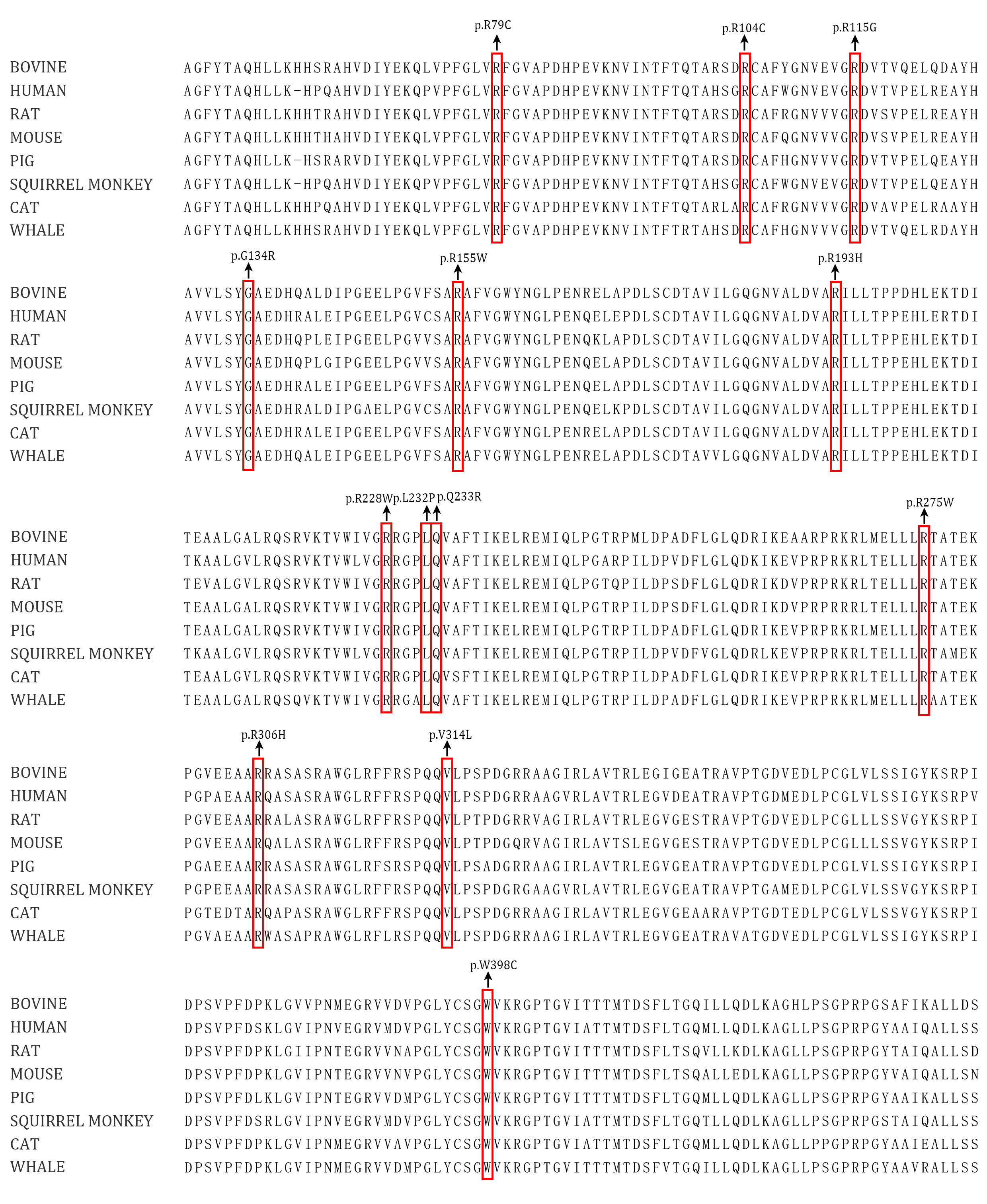

Supplement: Supplementary file 1 [file genes-14-00952-s001.zip › YIST-FDXR-Supplementary Figure S1.tif]
